# Omeprazole Treatment Failure in Gastroesophageal Reflux Disease and Genetic Variation at the *CYP2C* Locus

**DOI:** 10.3389/fgene.2022.869160

**Published:** 2022-05-19

**Authors:** Ping Siu Kee, Simran D. S. Maggo, Martin A. Kennedy, Murray L. Barclay, Allison L. Miller, Klaus Lehnert, Maurice A. Curtis, Richard L. M. Faull, Remai Parker, Paul K. L. Chin

**Affiliations:** ^1^ Department of Pathology and Biomedical Science, University of Otago, Christchurch, New Zealand; ^2^ Department of Medicine, University of Otago, Christchurch, New Zealand; ^3^ Department of Clinical Pharmacology, Christchurch Hospital, Christchurch, New Zealand; ^4^ Faculty of Science, University of Auckland, Auckland, New Zealand; ^5^ Faculty of Medical and Health Sciences, University of Auckland, Auckland, New Zealand

**Keywords:** GERD, refractory, CYP2C:TG, reflux, omeprazole, CYP2C19, ultrarapid metabolizer, pharmacogenetics

## Abstract

Omeprazole is extensively used to manage gastroesophageal reflux disease (GERD). It is primarily metabolized by CYP2C19. The CYP2C19*17 (rs12248560) allele and the recently described *CYP2C:TG* haplotype (rs11188059 and rs2860840) are associated with increased enzymatic activity, and may reduce omeprazole exposure. This observational study aimed to investigate the association between these genetic variants and omeprazole treatment failure in GERD. We recruited predominantly New Zealand European GERD patients who either did not respond to omeprazole or experienced breakthrough heartburn symptoms despite at least 8 weeks of omeprazole (≥40 mg/day). The GerdQ score was used to gauge symptomatic severity. A total of 55 cases were recruited with a median age (range) of 56 years (19–82) and GerdQ score of 11 (5–17). Of these, 19 (34.5%) were CYP2C19*17 heterozygotes and two (3.6%) were CYP2C19*17 homozygotes. A total of 30 (27.3%) *CYP2C:TG* haplotypes was identified in our cohort, with seven (12.7%) *CYP2C:TG* homozygotes, and 16 (29%) *CYP2C:TG* heterozygotes. No significant differences were observed for overall CYP2C19*17 alleles, CYP2C19*17/*17, overall *CYP2C:TG* haplotypes, and *CYP2C:TG* heterozygotes (*p* > 0.05 for all comparisons). Gastroscopy and 24-h esophageal pH/impedance tests demonstrated objective evidence of GERD in a subgroup of 39 (71%) cases, in which the *CYP2C:TG/TG* was significantly enriched (*p* = 0.03) when compared with the haplotype frequencies in a predominantly (91%) New Zealand European reference population, but not the CYP2C19*17/*17 (*p* > 0.99), when compared with the allele frequencies for the non-Finnish European subset of gnomAD. We conclude that omeprazole treatment failure in GERD is associated with *CYP2C:TG/TG*, but not CYP2C19*17.

## 1 Introduction

Symptomatic gastroesophageal reflux disease (GERD) has a global prevalence of around 14% (Nirwan et al., 2020). The mainstay treatment for GERD is proton-pump inhibitors (PPIs), which bind to the hydrogen-potassium ATPase enzyme in the parietal cells and inhibit gastric acid secretion (Mossner and Caca, 2005; Strand et al., 2017). However, PPI treatment failure remains a clinical challenge, with up to 45% of patients being refractory to treatment (El-Serag et al., 2010). Omeprazole is one of the most commonly prescribed PPIs globally. It is mainly metabolised by cytochrome P450 2C19 (CYP2C19) (Meyer, 1996).

Amongst clinically important *CYP2C19* genetic variants, CYP2C19*17 is associated with an increased CYP2C19 enzyme expression and activity (Sim et al., 2006) especially in homozygotes (ultrarapid metabolizers, UMs) (Sanford et al., 2013; Lima et al., 2021), with the reported mean omeprazole area under the curve (AUC) in UMs approximately half of the concentration observed in normal metabolizers (NMs) (CYP2C19*1/*1) (*p* = 0.04) (Baldwin et al., 2008). Furthermore, the pharmacokinetic variability associated with *CYP2C19* genotypes also affects the pharmacodynamics of omeprazole. Poor metabolizers (PMs) were shown to have a 12-fold higher mean omeprazole AUC and 2-fold higher intragastric pH compared to NMs, respectively (*p* = 0.0001 for both observations) (Furuta et al., 1999).

The current Clinical Pharmacogenetics Implementation Consortium (CPIC) guideline recommends a double daily dose of PPI for CYP2C19 UMs (defined by CYP2C19*17 homozygosity) (Lima et al., 2021), while the Dutch Pharmacogenetics Working Group (DPWG) recommended that CYP2C19 UMs should receive a three-fold higher omeprazole dose in *H. pylori* eradication therapy (Whirl-Carrillo et al., 2021). However, these recommendations were ranked as optional for CPIC, and minor clinical relevance by DPWG, likely due to the moderate or weak quality of existing evidences for this drug-gene interaction (Lima et al., 2021; Whirl-Carrillo et al., 2021).

In addition to the *17 allele, a recently described *CYP2C*:*TG* haplotype defined by two single nucleotide polymorphisms (SNPs) (rs2860840 C > T and rs11188059 G > A) is also associated with the CYP2C19 ultrarapid phenotype (Bråten et al., 2021). Herein we investigate the association of ultrarapid metabolism induced by CYP2C19*17 and *CYP2C*:*TG* haplotypes with omeprazole treatment failure in refractory GERD.

## 2 Materials and Methods

This is a sub-study of Understanding Adverse Drug Reactions using Genomic Sequencing (UDRUGS) (Maggo et al., 2017), which has ethical approval from the New Zealand (NZ) Health and Disability Ethics Committees (HDEC URA/11/11/065). The conduct of this sub-study was overseen by a gastroenterologist.

### 2.1 Study Participants

Screening and recruitment were carried out during September 2020—July 2021. Cases were recruited from referrals to the Department of Gastroenterology, Christchurch Hospital, Christchurch, NZ. All clinical data and information were obtained after informed consent, from health records and direct communication with the participants.

In this study, the case inclusion criteria were the presence of persistent heartburn (“burning feeling behind the breastbone”) and previous trials of omeprazole treatment at ≥ 40 mg total daily dose for a minimum of 8 weeks (Zerbib et al., 2021). Other symptoms indicative of acid reflux included acid brash and a metallic taste in the mouth. Participants who presented with heartburn less than 4 days in a week were only included if they had a GerdQ score of ≥8 (Jones et al., 2009). The GerdQ is a symptom-based scoring tool that measures symptom severity (see Supplementary Data Sheet Methods) (Jones et al., 2009). Participants were excluded if the GERD symptoms were diagnosed as secondary to other underlying clinical conditions, such as calcinosis, Raynaud phenomenon, esophageal dysmotility, sclerodactyly, telangiectasia (CREST) syndrome, eosinophilic esophagitis or motility disorder.

The highest daily dose of omeprazole trialled by each participant, and the list of selected concomitant medications known as risk factors for GERD (Lazenby et al., 2002; Mungan and Pinarbasi Simsek, 2017; UpToDate Inc, 2021) during treatment failure were recorded. All participants were asked to rate the efficacy of omeprazole in providing relief for their reflux symptoms. The rating ranged from 0% (never worked), 25% (hardly worked), 50% (only worked half of the time), 75% (almost resolved) to 100% (totally resolved).

Objective GERD evidence was determined on the basis of prior gastroscopy and/or pH-impedance monitoring tests. The definitions of positive pathological GERD included 24-h esophageal pH of <4 for ≥ 4.2% of the study time without PPI treatment, ≥ 1.2% with PPI treatment, or close correlation between reported heartburn symptoms and detected reflux events during pH/impedance monitoring. Study participants who presented with typical GERD symptoms in addition to having at least one positive pathological GERD test were categorized as cases with objective GERD evidence.

Participants were genotyped for CYP2C19*2 (rs4244285), CYP2C19*3 (rs4986893), CYP2C19*4 (rs28399504), CYP2C19*7 (rs72558186), CYP2C19*17 (rs12248560) alleles, SNPs rs2860840 C>T, and rs11188059 G>A. Each participant was assigned a CYP2C19 genotype-inferred phenotype, as per the CPIC guidelines (Hicks et al., 2017).

### 2.2 Sampling and Deoxyribonucleotide (DNA) Extraction

Samples for DNA were collected using blood (4ml x 3 ethylenediaminetetraacetic acid tubes) or saliva (Oragene^®^ DNA OG-500 kit-DNA Genotek Inc., Ottawa, Canada). DNA was extracted from saliva samples as per the manufacturer’s protocol (DNA Genotek Inc., 2021) while for blood samples, extraction was carried out as previously described (Miller et al., 1988; Maggo et al., 2019).

### 2.3 Polymerase Chain Reaction (PCR)

Oligonucleotide primers (Integrated DNA Technologies, Pte. Ltd., Singapore) used in this study are as listed in Supplementary Table S1. A 10 µl PCR reaction was prepared with 1X TAQ-Ti PCR buffer (Fisher Biotec), 0.2 mmol/L of each dNTP, 1.5 mmol/L MgCl_2_, 0.5 µmol/L of each primer, 1 mol/L betaine, 0.25 units of TAQ-Ti DNA polymerase (Fisher Biotec), and genomic DNA (20–100 ng/µl).

#### 2.3.1 *CYP2C19* Alleles

PCR was run using a touchdown protocol starting with incubation at 94°C for 2 min, followed by 15 cycles of 94°C for 15 s, 65°C for 15 s and 72°C for 1.5 min with an annealing temperature decrease of 1°C per cycle, followed by 20 cycles of 94°C for 15 s, 50°C for 15 s and 72°C for 1.5 min, and final extension at 72 ° C for 5 min. The cycle ended at 25°C for 2 min.

#### 2.3.2 *CYP2C*:*TG* Haplotype (rs2860840 and rs11188059)

The touchdown protocol (as described above) was used for SNP rs2860840, while an optimized PCR protocol was used to amplify SNP rs11188059: 94°C for 2 min, 35 cycles of 94°C for 15 s, 55°C for 15 s, and 72°C for 1.5 min. The cycle ended at 25°C for 2 min.

### 2.4 Sanger Sequencing

After PCR products were confirmed on a 1% agarose gel, the remaining PCR products were diluted in a 1:3 ratio in preparation for Sanger sequencing, run using BigDye^®^ Terminator v3.1 on 3130XL Genetic Analyzer.

### 2.5 Bioinformatic Analyses

Generated chromatograms were analysed on Geneious Prime Version 2020.1 (Biomatters Ltd. Auckland, NZ). *CYP2C19* reference gene sequence (NC_000010.11: 94664681-94855547), including the positions of all targeted SNPs, was downloaded from the National Centre for Biotechnology Information (NCBI) (https://www.ncbi.nlm.nih.gov/gene). Cases were genotyped by aligning the sequencing results with the annotated reference. CYP2C19*1 allele was assigned when no *CYP2C19* variations were observed.

### 2.6 *CYP2C*:*TG* Haplotypes From Reference Cohorts With Genomic Data

Three haplotypes were previously observed for the region encompassing rs2860840 (C > T) and rs11188059 (G > A), and these are referred to as TA, TG, and CG (Bråten et al., 2021). Estimated European haplotype frequencies, from the 1000 Genomes database, for haplotypes TA, TG, and CG were 12.2%, 19.6%, and 68.3%, respectively (Bråten et al., 2021). As this was recently discovered, validating the reported haplotype frequencies in the NZ European population was necessary. We examined these frequencies in two local NZ European cohorts with previously acquired unphased whole-genome sequencing (WGS) data (Macrogen, Inc. South Korea), or imputed and phased genome-wide genotyping data derived from the Infinium Global Screening (GSA) array (Illumina Inc. San Diego, United States).

Reference Cohort 1 (*N* = 179) was comprised of two related studies by our laboratory called UDRUGS (Maggo et al., 2017), and Genomic Analysis of Adverse Drug Reactions (GAARD) (Liau *et al.* manuscript in preparation). This included 38 samples with WGS data and 141 samples with imputed GSA genotyping array data, mapped to GRCh37. The WGS cohort was 95% NZ European, and the GSA genotyping array cohort was 89% NZ European. Overall, 91% (*N* = 179) of Reference Cohort 1 were NZ Europeans.

Reference Cohort 2 (*N* = 129) consisted of 120 samples from the Neurological Foundation Human Brain Bank at the Centre for Brain Research, University of Auckland (HDEC 14/NTA/208), and nine publicly available samples from the 1000 Genomes Project including Centre d'Etude du Polymorphisme Humain pedigree 1463 (Eberle et al., 2017), mapped to GRCh38. Approximately 97% of the 120 Brain Bank samples were NZ Europeans, while the nine publicly available samples were of well-established Caucasian ancestry.

Haplotype information derived from these two reference cohorts is detailed in Supplementary Table S2.

### 2.7 Statistical Analyses

Fisher’s exact tests were used to compare the cases with reference data sets, by grouping in the following ways: 1) Total number of CYP2C19*17 alleles, 2) CYP2C19*17 homozygotes, 3) Total number of *CYP2C*:*TG* haplotypes, 4) *CYP2C:TG* heterozygotes, and 5) *CYP2C:TG* homozygotes. In addition, subgroup analysis was conducted to evaluate the robustness of any detected association.

Comparisons of CYP2C19*17 alleles were made against the expected population frequency for non-Finnish European cohort (*N* = 7,672) of the Genome Aggregation Database (gnomAD) (Karczewski et al., 2020), with small study on NZ Europeans showing a comparable CYP2C19*17 allele frequency to gnomAD (Maggo et al., 2019).

*CYP2C:TG* haplotypes were compared with the predominantly NZ European Reference Cohort 1 (*N* = 179) because this haplotype requires the assessment of two SNPs. Statistical tests were conducted using GraphPad Prism (version 9.2.0, 332) and MedCalc^®^ (MedCalc Software Ltd, 2021).

A *p*-value of <0.05 was considered as statistically significant. Multiple comparison testing was not carried out for two reasons. First, omeprazole is mainly metabolised by CYP2C19 (Meyer, 1996), and the investigated *CYP2C* locus and *CYP2C19* gene are both known to affect CYP2C19 enzyme function. Both CYP2C19*17 allele and *CYP2C:TG* haplotype were associated with an increased metabolism phenotype in CYP2C19 (Sim et al., 2006; Bråten et al., 2021). Second, a single SNP (CYP2C19*17) and a haplotype (*CYP2C:TG*) were investigated for association with the targeted phenotype.

## 3 Results

### 3.1 Case Identification and Recruitment

A total of 189 study invitations were despatched. Out of the 122 (65%) who responded, 85 agreed to participate, and 67 were eligible for the study. Out of the 67 eligible cases, 12 were lost to follow-up or participants decided to withdraw from the study. Overall, a total of 55 cases were enrolled, with 33 blood and 22 saliva samples collected. A majority of the recruited cases were NZ Europeans (95%). Table 1 summarizes the demographic and clinical information, while more detail on individual cases can be found in Supplementary Table S3.

**TABLE 1:** Summary of the recruited cases (median, range).

Variables	Total cases (*N* = 55)
Sex (count): Female	47 (85%)
Age (years)	56 (19–82)
Ethnicity	
•New Zealand European	52 (94.5%)
•New Zealand Māori	2 (3.6%)
•Asian	1 (1.8%)
Weight (kg)	81 (46.4–161.0)
Body Mass Index (kg/m^2^)	30.1 (18.1–53.8)
^a^Concomitant medications (count)	
•Aspirin	9 (16.4%)
•Calcium channel blockers	8 (14.5%)
•High anticholinergics potency	4 (7.3%)
•Hypnotics and benzodiazepine	6 (10.9%)
•Nitrates	4 (7.3%)
•Non-steroidal anti-inflammatory drugs (NSAIDS)	5 (9.1%)
•Oral corticosteroid	4 (7.3%)
•Oestrogen replacement therapy	6 (10.9%)
•Tricyclic antidepressant	5 (9.1%)
GerdQ score	11^b^(5–17)
Objective ^c^GERD evidence (count)	39 (71%)
Rated omeprazole efficacy (count)	
•0%	14 (25.5%)
•25%	16 (29%)
•50%	22 (40%)
•75%	3 (5.5%)
The highest daily dose of omeprazole trialled (count)	
•40 mg	12 (22%)
•60 mg	3 (5.5%)
•80 mg	37 (67%)
•>80 mg	^d^3 (5.5%)
The highest daily dose of omeprazole trialled (mg)	
•Overall	80 (40–200)
•With objective ^c^GERD evidence	80 (40–200)
•Without objective ^c^GERD evidence	80 (40–160)

a Medications which were reported as a risk factor in the development of GERD.

b “0” was scored for one laryngopharyngeal reflux case.

c GERD: Gastroesophageal reflux disease.

d 120 mg, 160 mg, and 200 mg.

### 3.2 Genotype and Haplotype

**FIGURE 1:**
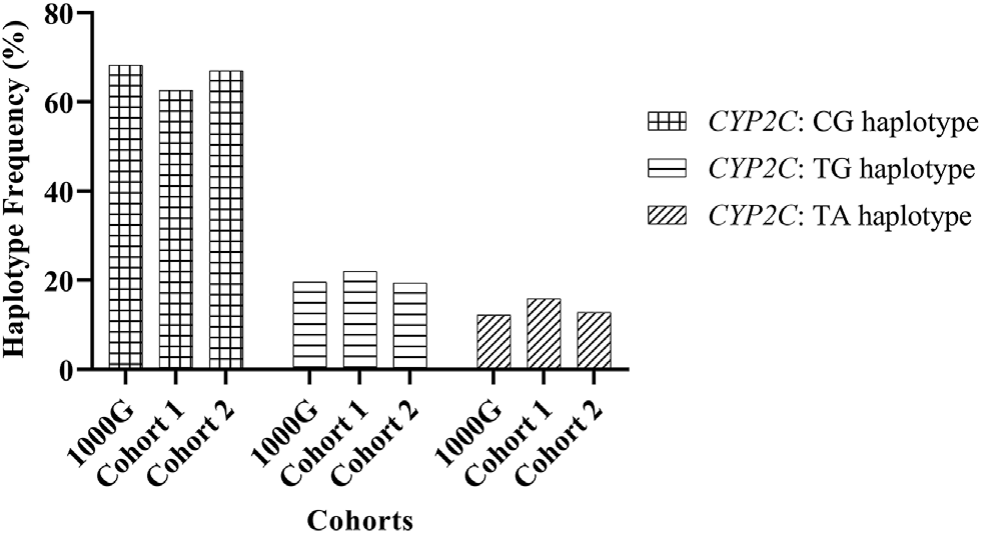
Frequency of *CYP2C*:haplotypes inferred from genetic variations rs2860840 C > T and rs11188059 G > A.

The observed frequency of TA, TG, and CG haplotypes in Reference Cohort 1 and Reference Cohort 2 were similar to data from the 1000 Genomes database (Figure 1), indicating an equivalent distribution of the haplotype frequency for the NZ European population.

With the absence of “CA” haplotype in Europeans (Bråten et al., 2021), diplotype TA/CG was assigned to cases who were heterozygotes for both SNPs (rs11188059 and rs2860840). The genotype results of CYP2C19*2 (rs4244285), CYP2C19*17 (rs12248560), rs2860840 (C > T), and rs11188059 (G > A) were available for both Reference Cohorts 1 and 2, with additional CYP2C19*3 (rs4986893) data for Reference Cohort 1 and CYP2C19*4 (rs28399504) data for Reference Cohort 2 (Supplementary Table S4-Excel File).

On the other hand, the genotypes for all the study cases included CYP2C19*2 (rs4244285), CYP2C19*3 (rs4986893), CYP2C19*4 (rs28399504), CYP2C19*7 (rs72558186) CYP2C19*17 (rs12248560), SNPs rs2860840 (C > T), and rs11188059 (G > A) (Supplementary Table S5).

#### 3.2.1 CYP2C19*17 Allele Comparisons Between Cases and Reference Population

Table 2 shows the counts of CYP2C19*17 alleles and CYP2C19*17/*17 (UMs) in the cases. A total of 23 (21%) CYP2C19*17 alleles were found in our cases (*N* = 55), with 19 (34.5%) heterozygotes and two homozygotes (3.6%). When compared with the frequencies in gnomAD non-Finnish Europeans (CYP2C19*17 alleles-23%, CYP2C19 UM-5.4%) (Karczewski et al., 2020), no significant differences were found for overall CYP2C19*17 allele and CYP2C19*17 homozygotes in overall cases and across subgroups.

**TABLE 2:** Group comparison of genotype and diplotype of CYP2C19*17 between reference population and cases (overall and within subgroups).

Group/Reference	Total (*N* = 55)		With objective ^a^GERD Evidence (*N* = 39)		Without objective ^a^GERD Evidence (*N* = 16)		Reference population, ^b^gnomAD (*N* = 7,672)	
Observations (allele and diplotype)	Total *17	*17 homo-zygotes	Total *17	*17 homo-zygotes	Total *17	*17 homo-zygotes	Total *17	*17 homo-zygotes
Count observed	23	2	20	2	3	0	3551	416
Total count	110	55	78	39	32	16	15344	7672
Frequency (%)	21.0	3.6	25.6	5.13	9.4	0	23.0	5.4
^c^ *p*-value	0.65	0.77	0.59	>0.99	0.09	>0.99	N/A	N/A

^a^Gastroesophageal reflux disease.

b Genome Aggregation Database.

c Compared with the reference population.

#### 3.2.2 *CYP2C:TG* Haplotype Comparisons Between Cases and Reference Population

The count of *CYP2C:TG* haplotypes, *CYP2C:TG* heterozygotes and *CYP2C:TG* homozygotes identified in the cases is as shown in Table 3. A comparison against Reference Cohort 1 showed no significant difference for all observations except for *CYP2C:TG* homozygotes. The frequency of these homozygotes was significantly enriched in cases with objective GERD evidence (*p* = 0.03), but not those without (*p* = 0.58) and overall cases (*p* = 0.06).

**TABLE 3:** Group comparison of haplotype and diplotype for CYP2C:TG between reference population and cases (overall and within subgroups).

Group/Reference	Total (*N* = 55)			With objective ^a^GERD Evidence (*N* = 39)			Without objective ^a^GERD Evidence (*N* = 16)			Reference population, cohort 1 (*N* = 179)		
Observations (haplotype and diplotype)	Total TG	TG hetero-zygotes	TG homo-zygotes	Total TG	TG hetero-zygotes	TG homo-zygotes	Total TG	TG hetero-zygotes	TG homo-zygotes	Total TG	TG hetero-zygotes	TG homo-zygotes
Count observed	30	16	7	22	10	6	8	6	1	76	58	9
Total count	110	55	55	78	39	39	32	16	16	358	179	179
Frequency (%)	27.3	29	12.7	28.2	25.6	15.4	25	37.5	6.25	21.2	32.4	5.0
^b^ *p*-value	0.19	0.74	0.06	0.18	0.45	*0.03	0.65	0.78	0.58	N/A	N/A	N/A

a Gastroesophageal reflux disease.

b Compared with the reference population.

*p-value < 0.05.

#### 3.2.3 *CYP2C19* Genotype-Inferred Phenotype Comparisons Between Cases and Reference Population

Our case cohort consisted of 15 (27%) intermediate, 24 (43.6%) normal, 14 (25%) rapid and two (3.6%) ultrarapid metabolizers (Supplementary Table S5). Out of the seven identified *CYP2C:TG* homozygotes, all were inferred to be normal metabolizers except for one case, who was a CYP2C19*4 heterozygote, leading to assignment of an intermediate metabolizer phenotype. Four out of the 16 *CYP2C:TG* heterozygotes were found with one non-functioning CYP2C19*2 allele.

On the other hand, the two ultrarapid metabolizers (CYP2C19*17/*17) in the cohort were *CYP2C:CG* homozygotes, while five out of the 19 CYP2C19*17 heterozygotes were CYP2C19*2 allele carriers.

## 4 Discussion

In this retrospective study, the case cohort with GERD refractory to omeprazole treatment ≥40 mg/day for a minimum of 8 weeks showed an association with *CYP2C:TG* homozygotes (TG/TG), but not CYP2C19*17 homozygotes (UMs), *CYP2C:TG* heterozygotes, overall CYP2C19*17 alleles, or overall *CYP2C:TG* haplotypes.

### 4.1 CYP2C19*17 Allele

Despite the extensive literature on the impact of *CYP2C19* genotypes on omeprazole pharmacokinetics, studies investigating the clinical efficacy of omeprazole with CYP2C19 rapid metabolizers (RMs) (*1/*17) and UMs (*17/*17) remain limited and were mostly carried out in non-GERD samples (Arévalo Galvis et al., 2019; Chwiesko et al., 2016; Molina-Infante et al., 2015).

Our non-significant findings (*p* > 0.05) with CYP2C19*17 alleles are consistent with a randomized prospective study on 50 samples with non-variceal upper-gastrointestinal bleeding (Chwiesko et al., 2016). The subjects were randomized to either 40 mg intravenous omeprazole bolus injection every 12 h or 8 mg/h continuous intravenous infusion for 72 h after an 80 mg intravenous omeprazole bolus dose. Genotyping and measuring the percentage of time with pH values >4.0 and >6.0 at several time points showed no significant association between CYP2C19 RM + UM and intragastric pH in both groups (Chwiesko et al., 2016).

In contrast, a case-control study on 34 children who underwent anti-reflux surgery after failing PPI therapy showed a significant over-representation of RM + UM in the cases (*p* = 0.035), when compared with the healthy control cohort (*N* = 457). However, this study was conducted on a paediatric population, who may have different GERD-related pathology compared to adults, and the cases were trialled on different types of PPI. Further, CYP2C19 RMs and UMs were grouped for analysis, and *CYP2C:TG* haplotype was not studied (Franciosi et al., 2018b). Separately, another study on 74 children who underwent esophageal pH testing while being treated with a PPI also showed that CYP2C19 RM + UM was associated with a longer (*p* = 0.03) and higher (*p* = 0.04) percentage of time in pH probe acid reflux outcomes of pH < 4.0 when compared with the controls (defined by the presence of at least one *CYP2C19* loss-of-function allele) (Franciosi et al., 2018a).

### 4.2 *CYP2C:TG* Haplotyp*e*

The CYP2C19 ultrarapid phenotype inferring *CYP2C:TG* haplotype is a novel finding from a detailed genetic study on escitalopram, another CYP2C19 substrate (Bråten et al., 2021). Besides *CYP2C:TG*, other haplotypes observed with the two SNPs (rs2860840 C > T and rs11188059 G > A) include CG and TA. The haplotype CA was not apparent in both the 1000 Genomes Project database (Bråten et al., 2021) and our Reference Cohorts. This indicated that the CA haplotype is either absent or very rare in Europeans. Using next-generation sequencing and phenotype quantification analysis on rs2860840 C > T and rs11188059 G > A, Bråten *et al.* (2021) found that *CYP2C:TG* heterozygotes and homozygotes were significantly associated with 16.7% (*p* < 0.001) and 24.8% (*p* = 0.0084) lower predicted escitalopram concentrations respectively, when compared with baseline (Bråten et al., 2021). In our refractory GERD cases, we found a significant (*p* = 0.03) association between *CYP2C:TG* homozygotes (TG/TG) and cases with objective evidence of GERD. Presumably, the less robust phenotypes from cases lacking objective evidence may explain the non-significant association with TG/TG within the subgroup and overall cases. These observations suggest that phenotype definition was crucial for the detection of this association.

### 4.3 *CYP2C19* Alleles and *CYP2C:TG* Haplotyp*e*

Bråten *et al.* (2021) suggested that CYP2C19*17 and the “T” allele of SNP rs2860840 were mutually exclusive (Bråten et al., 2021), and this was supported by our analysis. We found that CYP2C19*17 was absent in all *CYP2C:TG* homozygotes identified in both our cases and in the 141 phased GSA array data from Reference Cohort 1.

For other *CYP2C19* alleles, we observed a simultaneous presence of non-functioning *CYP2C19* (e.g., *2, *4) alleles and the two activity-enhancing genetic variations (e.g., CYP2C19*17, *CYP2C:TG*) studied in our cases. As the haplotype relationships between these SNPs cannot be directly ascertained due to their physical separation, we inferred the phasing of CYP2C19*2 with either CYP2C19*17 or the “T” allele of SNP rs2860840, by using the 141 phased GSA array data (Reference Cohort 1). All of the observed *2 variants were on the alternate alleles. However, one of the cases (RF1), is a CYP2C19*4 heterozygote who also carries two *CYP2C:TG* haplotypes. This highlights the possibility of having these genetic variations within the same haplotype. With regard to the functional impacts, Bråten *et al.* (2021) predicted an approximately 20% higher escitalopram mean serum concentration in samples with both *CYP2C:TG* and *CYP2C19* non-functioning alleles, when compared with baseline (Bråten et al., 2021). This indicated that the presence of *CYP2C:TG* haplotype may not be able to compensate for the null function alleles. Similarly, in simultaneous detection of CYP2C19*2 and CYP2C19*17 alleles, intermediate metabolizer phenotypes were suggested due to the dominant effect of CYP2C19*2 allele (de Vos et al., 2011; Hicks et al., 2017). Therefore, the presence of non-functioning *CYP2C19* alleles may have contributed to the non-significant associations reported with CYP2C19*17 and *CYP2C:TG* heterozygotes. Nonetheless, the impact of null function alleles in the presence of two metabolism enhancing genetic variations (e.g., case RF1) remains unclear.

## 5 Limitations

There are several limitations in the current study. First, this is a relatively small clinical study, which increases the risk of type II error and may have resulted in the non-significant (*p* > 0.05) CYP2C19*17 homozygote findings. However, the statistically significant *CYP2C:TG* haplotype finding, despite the small number of cases, highlights a potentially important finding that could be targeted by future pharmacogenetic research on PPI. Second, there is a degree of heterogeneity in the cases recruited. In addition to the three refractory GERD presentations suggested by the ESNM/ANMS consensus guideline (Zerbib et al., 2021), GERD is presented in several types of phenotypes such as erosive esophagitis and esophageal acid hypersensitivity (Aziz et al., 2016), where each phenotype has been reported to respond to PPIs differently (Kim et al., 2015; Mahoney and Rosen, 2019). The possible inclusion of different GERD phenotypes into our study may be a confounding factor. Third, other known risk factors of GERD such as concomitant medications (e.g., aspirin and oral corticosteroids) may have contributed partly to the refractory GERD. Fourth, part of this descriptive retrospective study was patient-recall dependent. Our study included omeprazole treatment failure cases that occurred up to 11 years prior to recruitment. Therefore, the risk of recall bias remains. Our last point concerns the generalizability of the study findings. While this study was carried out in a predominantly NZ European population, most NZ Europeans are of North-western European ancestry (Ministry of Culture and Heritage, 2008), which equates with the majority of the non-Finnish European population frequency in gnomAD (Karczewski et al., 2020). Therefore, our findings may generalise towards non-Finnish European populations in general.

## 6 Future Work and Conclusion

There are two suggestions for future research. First, a prospective study with pharmacokinetic data including plasma concentrations of omeprazole should be carried out. Second, use of more exclusive GERD phenotype definitions, based on objective measures, and including screening for individual responses towards esomeprazole, another major CYP2C19 substrate, would be useful. In regards to pharmacogenetics, the presence of undiscovered genetic variants within the *CYP2C* locus and other regions which may induce clinically relevant CYP2C19 ultrarapid phenotypes should be further explored.

In conclusion, this study of 55 GERD patients who experienced omeprazole treatment failure showed a significant association with CYP2C19 ultrarapid phenotype inferred by *CYP2C:TG/TG*, but not CYP2C19*17/*17. If this finding is independently verified in a larger study, it would suggest that a higher omeprazole dose may be helpful in refractory GERD cases carrying two *CYP2C*:TG haplotypes (TG/TG). While further research in larger cohorts is necessary to replicate these observations, our study provides a preliminary insight into the clinical application of pharmacogenetics in dosing and optimising omeprazole response.

## Data Availability

The original contributions presented in the study are publicly available. This data can be found here: https://github.com/Pharmacogenetecist/Omeprazole-treatment-failure-in-gastro-esophageal-reflux-disease-and-genetic-variation-at-the-CYP2C-/blob/main/Omeprazole2C19Data.

## Abbreviations

ASNM, American society for neurogastroenterology and motility; AUC, area under the curve; CPIC, clinical pharmacogenetics implementation consortium; CREST, calcinosis, raynaud phenomenon, esophageal dysmotility, sclerodactyly, telangiectasia; CYP, cytochrome P450; DNA, deoxyribonucleic acid; ESNM, European society for neurogastroenterology and Motility; GAARD, genomic analysis of adverse drug reactions; GERD, gastroesophageal reflux disease; GSA, global screening array; IM, intermediate metabolizer; NCBI, national centre for biotechnology information; NM, normal metabolizer; PCR, polymerase chain reaction; PM, poor metabolizer; PPI, proton-pump inhibitor; RM, rapid metabolizer; SNP, single nucleotide polymorphisms; UDRUGS, understanding adverse drug reactions using genomic sequencing; UM, ultrarapid metabolizer; WGS, whole-genome sequencing.
