# Supplementary material for: Omeprazole Treatment Failure in Gastroesophageal Reflux Disease and Genetic Variation at the CYP2C Locus
Source: Front Genet. 2022 May 19;13:869160. doi: 10.3389/fgene.2022.869160 (PMC9160307; doi:10.3389/fgene.2022.869160)
Supplement: Supplementary file 1 [file Table1.DOCX]

# **Supplementary Tables**

Supplementary Table 1 List of primers used in the study.

| **Primer Label** | **Sequence (5’-3’)** | **Targeted** ^†^**SNPs (* allele)** | **Amplicon size (base pairs)** |
| --- | --- | --- | --- |
| CYP2C19 EX5 *2 F | ATC AAT CAG GTT GTG CAA ACT CT | rs4244285 (*2), rs72558186 (*7) | 847 |
| CYP2C19 EX5 *2 R | TCT CAC TGG AAG CTG CAG AA |  |  |
| CYP2C19 EX4 *3 F | TGT GTT GAT TTT ATG CAT GCC A | rs4986893 (*3) | 691 |
| CYP2C19 EX4 *3 R | AAG TGT GAA TTG AAG GAC AAG CC |  |  |
| CYP2C19 *4F | CGG TGC ATT GGA ACC ACT TG | rs28399504 (*4) | 930 |
| CYP2C19 *4R | GTC CTA AAC CCA CAG CTG CT |  |  |
| CYP2C19 *17 F | CCA GGA GGT CAA GAA GCC TTA G | rs12248560 (*17) | 838 |
| CYP2C19 *17 R | AAG TGG TTC CAA TGC ACC GT |  |  |
| CYP2C18_rs059_S2_F | ATG TGG TCT TTG TTT GTC TGA CT | rs11188059 | 727 |
| CYP2C18_rs059_S2_R | AGA CAC TTG ACA AAA TGG GAG A |  |  |
| CYP2C18_rs840_S1_F | ATC CAG CCA TCC TTC CAG TC | rs2860840 | 769 |
| CYP2C18_rs840_S1_R | CCA GCA CAG CCT CTT CAC AT |  |  |

^†^Single Nucleotide Polymorphisms.
