# Supplementary material for: Omeprazole Treatment Failure in Gastroesophageal Reflux Disease and Genetic Variation at the CYP2C Locus
Source: Front Genet. 2022 May 19;13:869160. doi: 10.3389/fgene.2022.869160 (PMC9160307; doi:10.3389/fgene.2022.869160)
Supplement: Supplementary file 2 [file Table2.DOCX]

# **Supplementary Tables**

Supplementary Table 2 Number of observed haplotypes CYP2C: CG, TG, TA and CA.

| **Group** | **1000G** | | | | **Cohort 1 (N=179)** | | | | **Cohort 2 (N=129)** | | | |
| --- | --- | --- | --- | --- | --- | --- | --- | --- | --- | --- | --- | --- |
| **Haplotypes** | **CG** | **TG** | **TA** | **CA** | **CG** | **TG** | **TA** | **CA** | **CG** | **TG** | **TA** | **CA** |
| **Count observed** | 387 | 111 | 69 | 0 | 224 | 77 | 57 | 0 | 175 | 50 | 33 | 0 |
| **Percentage (%)** | 68.3 | 19.6 | 12.2 | 0 | 62.6 | 21.5 | 15.9 | 0 | 67.8 | 19.4 | 12.8 | 0 |
| **Total count** | 567 | | | | 358 | | | | 258 | | | |
