# Supplementary material for: Omeprazole Treatment Failure in Gastroesophageal Reflux Disease and Genetic Variation at the CYP2C Locus
Source: Front Genet. 2022 May 19;13:869160. doi: 10.3389/fgene.2022.869160 (PMC9160307; doi:10.3389/fgene.2022.869160)
Supplement: Supplementary file 3 [file Table5.DOCX]

# **Supplementary Tables**

Supplementary Table 5 Genotype and haplotype results for all cases (N=55).

| **Code** | ^†^***CYP2C19*2* (rs4244285)** | ^†^***CYP2C19*3* (rs4986893)** | ^†^***CYP2C19*4* (rs28399504)** | ^†^***CYP2C19*7* (rs72558186)** | ^†^***CYP2C19**17 (rs12248560)** | ^‡^**CYP2C19 genotype (inferred phenotype)** | ^†§^**SNP rs2860840 C>T** | ^†§^**SNP rs11188059 G>A** | **Diplotype for *CYP2C*: locus** |
| --- | --- | --- | --- | --- | --- | --- | --- | --- | --- |
| RF1 | 0 | 0 | 1 | 0 | 0 | *1/*4 (IM) | 2 | 0 | TG/TG |
| RF2 | 1 | 0 | 0 | 0 | 0 | *1/*2 (IM) | 0 | 0 | CG/CG |
| RF3 | 0 | 0 | 0 | 0 | 1 | *1/*17 (RM) | 1 | 0 | TG/CG |
| RF4 | 0 | 0 | 0 | 0 | 1 | *1/*17 (RM) | 0 | 0 | CG/CG |
| RF5 | 1 | 0 | 0 | 0 | 0 | *1/*2 (IM) | 1 | 1 | TA/CG |
| RF6 | 1 | 0 | 0 | 0 | 0 | *1/*2 (IM) | 1 | 0 | TG/CG |
| RF7 | 1 | 0 | 0 | 0 | 1 | *2/*17 (IM) | 0 | 0 | CG/CG |
| RF8 | 1 | 0 | 0 | 0 | 1 | *2/*17 (IM) | 0 | 0 | CG/CG |
| RF9 | 0 | 0 | 0 | 0 | 1 | *1/*17 (RM) | 0 | 0 | CG/CG |
| RF10 | 1 | 0 | 0 | 0 | 0 | *1/*2 (IM) | 1 | 0 | TG/CG |
| RF11 | 0 | 0 | 0 | 0 | 1 | *1/*17 (RM) | 1 | 1 | TA/CG |
| RF12 | 0 | 0 | 0 | 0 | 0 | *1/*1 (NM) | 2 | 0 | TG/TG |
| RF13 | 0 | 0 | 0 | 0 | 1 | *1/*17 (RM) | 1 | 0 | TG/CG |
| RF14 | 0 | 0 | 0 | 0 | 0 | *1/*1 (NM) | 2 | 0 | TG/TG |
| RF15 | 0 | 0 | 0 | 0 | 0 | *1/*1 (NM) | 1 | 0 | TG/CG |
| RF16 | 0 | 0 | 0 | 0 | 0 | *1/*1 (NM) | 2 | 1 | TG/TA |
| RF17 | 1 | 0 | 0 | 0 | 0 | *1/*2 (IM) | 1 | 1 | TA/CG |
| RF18 | 0 | 0 | 0 | 0 | 0 | *1/*1 (NM) | 2 | 1 | TG/TA |
| RF19 | 0 | 0 | 0 | 0 | 0 | *1/*1 (NM) | 1 | 1 | TA/CG |
| RF20 | 0 | 0 | 0 | 0 | 0 | *1/*1 (NM) | 2 | 0 | TG/TG |
| RF21 | 0 | 0 | 0 | 0 | 0 | *1/*1 (NM) | 1 | 0 | TG/CG |
| RF22 | 0 | 0 | 0 | 0 | 1 | *1/*17 (RM) | 1 | 1 | TA/CG |
| RF23 | 0 | 0 | 0 | 0 | 1 | *1/*17 (RM) | 1 | 1 | TA/CG |
| RF24 | 0 | 0 | 0 | 0 | 2 | *17/*17 (UM) | 0 | 0 | CG/CG |
| RF25 | 1 | 0 | 0 | 0 | 0 | *1/*2 (IM) | 0 | 0 | CG/CG |
| RF26 | 0 | 0 | 0 | 0 | 0 | *1/*1 (NM) | 2 | 1 | TG/TA |
| RF27 | 0 | 0 | 0 | 0 | 2 | *17/*17 (UM) | 0 | 0 | CG/CG |
| RF28 | 0 | 0 | 0 | 0 | 0 | *1/*1 (NM) | 2 | 1 | TG/TA |
| RF29 | 1 | 0 | 0 | 0 | 1 | *2/*17 (IM) | 0 | 0 | CG/CG |
| RF30 | 0 | 0 | 0 | 0 | 1 | *1/*17 (RM) | 1 | 1 | TA/CG |
| RF31 | 0 | 0 | 0 | 0 | 0 | *1/*1 (NM) | 1 | 1 | TA/CG |
| RF32 | 0 | 0 | 0 | 0 | 0 | *1/*1 (NM) | 2 | 0 | TG/TG |
| RF33 | 1 | 0 | 0 | 0 | 0 | *1/*2 (IM) | 1 | 1 | TA/CG |
| RF34 | 0 | 0 | 0 | 0 | 1 | *1/*17 (RM) | 1 | 0 | TG/CG |
| RF35 | 0 | 0 | 0 | 0 | 0 | *1/*1 (NM) | 1 | 0 | TG/CG |
| RF36 | 0 | 0 | 0 | 0 | 0 | *1/*1 (NM) | 0 | 0 | CG/CG |
| RF37 | 0 | 0 | 0 | 0 | 0 | *1/*1 (NM) | 0 | 0 | CG/CG |
| RF38 | 0 | 0 | 0 | 0 | 0 | *1/*1 (NM) | 0 | 0 | CG/CG |
| RF39 | 1 | 0 | 0 | 0 | 0 | *1/*2 (IM) | 1 | 0 | TG/CG |
| RF40 | 0 | 0 | 0 | 0 | 1 | *1/*17 (RM) | 1 | 1 | TA/CG |
| RF41 | 1 | 0 | 0 | 0 | 0 | *1/*2 (IM) | 1 | 0 | TG/CG |
| RF42 | 0 | 0 | 0 | 0 | 1 | *1/*17 (RM) | 0 | 0 | CG/CG |
| RF43 | 0 | 0 | 0 | 0 | 0 | *1/*1 (NM) | 0 | 0 | CG/CG |
| RF44 | 0 | 0 | 0 | 0 | 0 | *1/*1 (NM) | 1 | 1 | TA/CG |
| RF45 | 0 | 0 | 0 | 0 | 0 | *1/*1 (NM) | 2 | 2 | TA/TA |
| RF46 | 0 | 0 | 0 | 0 | 1 | *1/*17 (RM) | 0 | 0 | CG/CG |
| RF47 | 0 | 0 | 0 | 0 | 0 | *1/*1 (NM) | 1 | 1 | TA/CG |
| RF48 | 0 | 0 | 0 | 0 | 1 | *1/*17 (RM) | 0 | 0 | CG/CG |
| RF49 | 0 | 0 | 0 | 0 | 0 | *1/*1 (NM) | 2 | 1 | TG/TA |
| RF50 | 0 | 0 | 0 | 0 | 0 | *1/*1 (NM) | 2 | 0 | TG/TG |
| RF51 | 0 | 0 | 0 | 0 | 0 | *1/*1 (NM) | 2 | 0 | TG/TG |
| RF52 | 1 | 0 | 0 | 0 | 1 | *2/*17 (IM) | 0 | 0 | CG/CG |
| RF53 | 0 | 0 | 0 | 0 | 1 | *1/*17 (RM) | 1 | 1 | TA/CG |
| RF54 | 1 | 0 | 0 | 0 | 1 | *2/*17 (IM) | 0 | 0 | CG/CG |
| RF55 | 0 | 0 | 0 | 0 | 0 | *1/*1 (NM) | 1 | 0 | TG/CG |

^†^‘0’, ‘1’ and ‘2’ represented the number of alternative alleles carried by each case.

^‡^NM: normal metabolizer, IM: intermediate metabolizer, RM: rapid metabolizer, UM: ultrarapid metabolizer

^§^Single Nucleotide Polymorphisms.
