# Supplementary material for: Omeprazole Treatment Failure in Gastroesophageal Reflux Disease and Genetic Variation at the CYP2C Locus
Source: Front Genet. 2022 May 19;13:869160. doi: 10.3389/fgene.2022.869160 (PMC9160307; doi:10.3389/fgene.2022.869160)
Supplement: Supplementary file 5 [file Table3.DOCX]

# **Supplementary Tables**

Supplementary Table 3 Demographic and clinical information for individual cases.

| **Code** | **Sex** | ^†^**Ethnicity** | ^‡^**Age** | **Weight (kg)** | **Body Mass Index (kg/m^2^)** | **Highest omeprazole dose trialled (mg)** | ^§^**Gastro-scopy** | ^§^**Manometry** | **Total percentage time of esophageal acidification pH<4.0 (%)** | **GerdQ score** | **Rating on omeprazole efficacy (%)** | **With (+) or without (-) objective GERD evidences** |
| --- | --- | --- | --- | --- | --- | --- | --- | --- | --- | --- | --- | --- |
| RF1 | M | E | 66 | 96.1 | 33.3 | 80 | No record | No record | 6.3 | 10 | 0 | + |
| RF2 | F | E | 54 | 141.0 | 47.7 | 80 | 40cm large HH | Normal with slight incomplete relaxation of the lower esophageal sphincter | 11.1 | 5 | 50 | + |
| RF3 | F | E | 68 | 74.5 | 26.1 | 80 | No record | No record | No record | 10 | 25 | - |
| RF4 | F | E | 62 | 69.2 | 27.4 | 80 | Irregular Z line and inflammation | 1) TLOSRs  2) Risk of dysphagia  3) Small HH | ^¶^5.5 | 10 | 50 | + |
| RF5 | F | E | 69 | 84.0 | 29.4 | 80 | 1) Irregular Z line  2) 3cm HH  3) Gaping lower esophageal sphincter | Hypotensive lower esophageal sphincter with normal esophageal body motility | 21.3 | 10 | 50 | + |
| RF6 | F | M | 45 | 88.2 | 34.9 | 80 | Normal | Normal | 12.5 | 17 | 50 | + |
| RF7 | F | E | 76 | 67.0 | 25.5 | 200 | 1) 34-37cm HH  2) LA Grade B esophagitis | No record | No record | 11 | 25 | + |
| RF8 | F | E | 47 | 71.0 | 24.9 | 80 | 1.7cm HH | Frequent TLOSRs | 6.1 | 14 | 50 | + |
| RF9 | M | E | 69 | 113.0 | 37.3 | 60 | Widely patent Schatzki's ring | No record | 6.0 | 11 | 50 | + |
| RF10 | F | E | 74 | 62.0 | 23.1 | 80 | No record | No record | 4.5 | 11 | 50 | + |
| RF11 | F | E | 53 | 65.5 | 24.1 | 80 | 1) 32cm HH  2) Previous fundoplication wrap slipped into the chest | Poor peristaltic function | 7.3 | 9 | 0 | + |
| RF12 | F | E | 21 | 117.0 | 44.0 | 80 | 1) Medium-sized HH  2) LA Grade A esophagitis | 1) 1.9cm HH  2) Absent lower esophageal sphincter tone | 5.7 | 16 | 0 | + |
| RF13 | F | E | 54 | 72.5 | 21.4 | 80 | 1) 0.7cm HH  2) LA Grade A esophagitis | Normal | 7.0 | 14 | 0 | + |
| RF14 | F | E | 45 | 46.4 | 18.1 | 80 | Barrett's oesophagus 11cm | 1) Absent lower esophageal sphincter tone  2) Aperistalsis | 26.6 | 13 | 25 | + |
| RF15 | F | E | 44 | 82.0 | 32.0 | 80 | Normal | Frequent failed esophageal peristalsis | 4.3 | 10 | 25 | + |
| RF16 | F | E | 57 | 70.7 | 31.0 | 80 | No record | 1) TLOSRs  2) Poor lower esophageal sphincter tone  3) Intermittent failed peristalsis | 26.5 | 12 | 0 | + |
| RF17 | F | E | 38 | 88.0 | 30.4 | 40 | Normal | Normal | 11.1 | 10 | 50 | + |
| RF18 | F | E | 62 | 88.5 | 32.1 | 40 | Normal | Normal | 4.7 | 10 | 0 | + |
| RF19 | F | E | 50 | 90.0 | 32.3 | 120 | Normal | 1) TLOSRs  2) Weak peristalsis with small defects | 6.3 | 8 | 25 | + |
| RF20 | F | E | 61 | 76.0 | 25.7 | 80 | No record | Frequent weak peristalsis with absent lower esophageal sphincter tone | 11.2 | 11 | 50 | + |
| RF21 | F | E | 72 | 89.0 | 31.2 | 80 | 1) Small HH  2) Cystic fundic gland polyps | No record | No record | 9 | 25 | - |
| RF22 | F | E | 51 | 74.0 | 26.2 | 80 | No record | No record | 7.0 | 13 | 75 | + |
| RF23 | F | E | 53 | 82.7 | 33.6 | 80 | 1) Irregular Z line  2) HH | Poor function during bread swallows with dysphagia | 5.0 | 12 | 50 | + |
| RF24 | F | E | 56 | 62.5 | 23.0 | 80 | No record | No record | 21.1 | 12 | 35 | + |
| RF25 | M | E | 73 | 161.0 | 53.8 | 80 | Small sliding HH | Numerous TLOSRs | No record | 10 | 25 | - |
| RF26 | F | E | 55 | 81.2 | 29.8 | 40 | 2cm HH | No record | 26.2 | 13 | 50 | + |
| RF27 | F | E | 50 | 85.0 | 29.4 | 80 | No record. Summary from gastro clinic letter stated irregular squamocolumnar junction at 37cm with no active esophagitis or Barrett’s mucosa and a small HH. | 1) 0.9cm HH  2) Ineffective motility | 13.5 | 9 | 50 | + |
| RF28 | M | E | 76 | 89.0 | 30.1 | 80 | Normal with the patulous oesophagus | Slow peristalsis with solid food, within low normal range | 10.0 | 10 | 25 | + |
| RF29 | M | E | 19 | 57.5 | 18.4 | 40 | No record | 1) TLOSRs  2) 0.6cm HH | 5.7 | 6 | 0 | + |
| RF30 | F | E | 71 | 87.0 | 34.0 | 80 | Normal, with histological evidence of esophagitis | Probable TLOSRs | 1.2 | 16 | 25 | + |
| RF31 | M | E | 60 | 78.0 | 26.4 | 40 | No record | No record | 22.5 | 11 | 25 | + |
| RF32 | F | E | 68 | 66.0 | 23.4 | 40 | 1) Irregular Z line  2) Hiatus hernia resulting from slipped Nissen fundoplication  3) Esophageal candidiasis | 1) Normal wet swallows  2) Poor bread swallows | 31.3 | 9 | 50 | + |
| RF33 | F | E | 42 | 94.0 | 31.8 | 80 | Normal with diffuse erythematous or erosive duodenitis affecting D2 | Delay in lower esophageal sphincter relaxation | 11.8 | 12 | 25 | + |
| RF34 | F | E | 35 | 120.0 | 42.5 | 60 | 1) 5cm HH  2) LA grade A esophagitis | Ineffective motility with very lax lower esophageal sphincter | 5.3 | 12 | 50 | + |
| RF35 | F | E | 82 | 90.0 | 34.3 | 40 | Waitlisted | No record | No record | 11 | 50 | - |
| RF36 | F | A | 46 | 85.0 | 35.4 | 40 | No record | No record | No record | 9 | 50 | - |
| RF37 | F | E | 27 | 114.9 | 40.7 | 40 | Normal | No record | No record | 12 | 50 | - |
| RF38 | F | E | 60 | 62.0 | 21.5 | 40 | HH | No record | No record | 9 | 25 | - |
| RF39 | F | E | 32 | 63.2 | 20.9 | 160 | No record | No record | No record | 5 | 0 | - |
| RF40 | F | E | 66 | 104.5 | 42.4 | 40 | 1) Large HH  2) Irregular Z-line | No record | No record | 16 | 75 | - |
| RF41 | F | M | 59 | 94.0 | 35.8 | 80 | 1) 4cm HH  2) Gastric polyps | No record | No record | 9 | 50 | - |
| RF42 | F | E | 71 | 74.3 | 31.3 | 40 | 1) LA grade C erosive esophagitis  2) Medium-sized HH with Cameron's ulcer  3) Multiple fundic gland polyps | No record | No record | 12 | 0 | + |
| RF43 | F | E | 31 | 56.0 | 20.1 | 80 | Normal | No record | No record | 6 | 50 | - |
| RF44 | F | E | 59 | 77.0 | 30.1 | 60 | No record | No record | No record | 15 | 0 | - |
| RF45 | F | E | 62 | 66.6 | 25.7 | 80 | West Coast District Health Board endoscopy: normal | No record | 10.3 | 0 | 0 | + |
| RF46 | F | E | 49 | 72.0 | 29.2 | 80 | No record | Waitlisted | Waitlisted | 9 | 0 | - |
| RF47 | M | E | 72 | 102.0 | 30.8 | 80 | 1) Sliding medium-sized hiatus hernia  2) Incompetent lower esophageal sphincter | No record | No record | 15 | 50 | - |
| RF48 | M | E | 36 | 91.2 | 28.1 | 80 | Small HH | 50% normal peristalsis with wet swallows only | 4.3 | 11 | 0 | + |
| RF49 | F | E | 51 | 67.0 | 24.3 | 80 | Medium HH | No record | Waitlisted | 14 | 25 | - |
| RF50 | F | E | 41 | 68.5 | 23.4 | 80 | Gaping lower esophageal sphincter | Normal | 0.9 (6 out of 7 heartburn symptoms were associated with pH-detected reflux) | 12 | 75 | + |
| RF51 | F | E | 76 | 95.0 | 32.9 | 80 | 1) 4cm HH  2) Cystic fundic gland polyps (benign)  3) Mildly dilated lower oesophagus | No record | No record | 17 | 50 | - |
| RF52 | F | E | 47 | 98.7 | 37.1 | 80 | 1) LA grade B reflux esophagitis  2) Medium-sized HH | Reduced lower esophageal sphincter tone | No record | 12 | 25 | + |
| RF53 | F | E | 72 | 67.0 | 24.0 | 80 | 1) LA grade B reflux esophagitis  2) Small-sized HH  3) Irregular Z-line | No record | No record | 11 | 0 | + |
| RF54 | F | E | 51 | 80 | 28.0 | 80 | 1) LA grade A esophagitis  2) Medium-sized HH | 1) Ineffective motility with wet swallows but improved function during bread swallows.  2) Hypertensive upper esophageal sphincter | No record | 12 | 25 | + |
| RF55 | F | E | 64 | 77 | 31.2 | 80 | 1) LA grade C esophagitis  2) Small-sized HH | 1) Hypo-contractile disorder  2) TLOSRs  3) Little or no resting pressure in the lower esophageal sphincter | 5.5 | 10 | 50 | + |

^†^Ethnicity: New Zealand European (E), New Zealand Māori (M), and Asian (A).

^‡^Age at the timepoint of omeprazole treatment failure.

^§^Abbreviations: Hiatal hernia (HH) and transient lower esophageal sphincter relaxation (TLOSRs).

^¶^Procedure conducted with proton-pump inhibitor treatment.
