# Supplementary material for: Omeprazole Treatment Failure in Gastroesophageal Reflux Disease and Genetic Variation at the CYP2C Locus
Source: Front Genet. 2022 May 19;13:869160. doi: 10.3389/fgene.2022.869160 (PMC9160307; doi:10.3389/fgene.2022.869160)
Supplement: Supplementary file 6 [file DataSheet1.DOCX]

# **Supplementary Method**

## GerdQ questionnaire

**SCREENING FORM**

**Why are reflux symptoms not well controlled by medicines in some patients? – Omeprazole Treatment Failure**

Name:

Kindly tick (√) or fill in the appropriate box.

Section A: Medication History

| No | Questions | Answers | | | | |
| --- | --- | --- | --- | --- | --- | --- |
| 1 | Are you currently or have you previously been prescribed omeprazole (Losec)? | Yes | | | | No |
|  |  |  | | | |  |
| 2 | Dose of omeprazole prescribed | 10 mg | 20 mg | | 40 mg | Other (Please specify): |
|  |  |  |  | |  |  |
| 3 | Frequency of omeprazole prescribed | Once daily | | Twice daily | | Other (Please specify): |
|  |  |  | |  | |  |
| 4 | Duration of omeprazole prescribed (e.g. 8 weeks) |  | | | | |

Section B: Describing the symptoms you experience (or experienced) **in a typical week** when you are (or were) taking omeprazole treatment.

| No | Questions | 0 day | 1 day | 2-3 days | | 4-7 days | |
| --- | --- | --- | --- | --- | --- | --- | --- |
| 1 | How often did you have a burning feeling behind your breastbone (heartburn)? | 0 | 1 | 2 | | 3 | |
| 2 | How often did you have stomach contents (liquid or food) moving upwards to your throat or mouth (regurgitation)? | 0 | 1 | 2 | | 3 | |
| 3 | How often did you have pain in the centre of the upper stomach? | 3 | 2 | 1 | | 0 | |
| 4 | How often did you have nausea? | 3 | 2 | 1 | | 0 | |
| 5 | How often did you have difficulty getting a good night’s sleep because of your heartburn and/or regurgitation? | 0 | 1 | 2 | | 3 | |
| 6 | How often did you take additional medication for your heartburn and/or regurgitation, other than prescribed (e.g. Gaviscon, Mylanta, Quick-Eze, Acidex and Titralac)? | 0 | 1 | 2 | | 3 | |
| 7 | How much do you agree with the statement “Omeprazole provided relief from my reflux symptoms”:  100% (Totally resolved)  75% (Almost resolved)  50% (Only works, half of the time)  25% (Hardly worked)  0% (Never worked) | 100% | 75% | 50% | 25% | | 0% |
|  |  |  |  |  |  | |  |

The GerdQ score consists of four positive (questions 1, 2, 5, and 6) and two negative (questions 3 and 4) predictors of GERD. Point values were added for each corresponding answer. A cut off score of ≥ 8 was used in this study.

Thank you.
